# Exploring voice as a digital phenotype in adults with ADHD

**DOI:** 10.1038/s41598-025-01989-x

**Published:** 2025-05-24

**Authors:** Georg G. von Polier, Eike Ahlers, Julia Volkening, Jörg Langner, Kaustubh R. Patil, Simon B. Eickhoff, Florian Helmhold, Agnieszka Ewa Krautz, Daina Langner

**Affiliations:** 1 Institute of Neuroscience and Medicine Brain and Behaviour, Forschungszentrum Jülich, Wilhelm-Johnen-Str, 52528 Jülich, Germany; 2 Clinic for Child and Adolescent Psychiatry, Psychotherapy and Psychosomatics, University Leipzig, Leipzig, Germany; 3 Clinic for Child and Adolescent Psychiatry, Psychosomatics and Psychotherapy, RWTH Aachen University, Aachen, Germany; 4 Institute of Psychiatry Campus Benjamin Franklin, Charité – Universitätsmedizin Berlin, corporate member of Freie Universität Berlin, Humboldt-Universität zu Berlin, Berlin Institute of Health, Berlin, Germany; 5 Clinic for Psychiatry, Psychotherapy and Psychosomatic medicine, Johanniter-Krankenhaus Treuenbrietzen, Treuenbrietzen, Germany; 6 PeakProfiling GmbH, Berlin, Germany; 7 Institute of Systems Neuroscience, Medical Faculty, Heinrich Heine University Düsseldorf, Düsseldorf, Germany; 8 Institute of Medical Psychology and Behavioral Neurobiology, University of Tübingen, Tübingen, Germany

**Keywords:** ADHD, Voice as a biomarker, AI diagnostics, Digital biomarker, Machine learning, Precision psychiatry, Machine learning, Diagnostic markers, ADHD

## Abstract

Current diagnostic procedures for attention deficit hyperactivity disorder (ADHD) are mainly subjective and prone to bias. While research on potential biomarkers, including EEG, brain imaging, and genetics is promising, it has yet to demonstrate clinical utility. Dopaminergic signaling alternations and executive functioning, crucial to ADHD pathology, are closely related to voice production. Consistently, previous studies point to alterations in voice and speech production in ADHD. However, studies investigating voice in large clinical samples allowing for individual-level prediction of ADHD are lacking. Here, 387 ADHD patients, 204 healthy controls, and 100 psychiatric controls underwent standardized diagnostic assessment. Subjects provided multiple 3-minutes speech samples, yielding 920 samples. Based on prosodic voice features, random forest-based classifications were performed, and cross-validated out-of-sample accuracy was calculated. The classification of ADHD showed the best performance in young female participants (AUC = 0.87) with lower performance in older participants and males. Psychiatric comorbidity did not alter the classification performance. Voice features were associated with ADHD-symptom severity as indicated by random forest regressions. In summary, prosodic features seem to be promising candidates for further research into voice-based digital phenotypes of ADHD.

**Supplementary Information:**

The online version contains supplementary material available at 10.1038/s41598-025-01989-x.

## Introduction

Attention Deficit Hyperactivity Disorder (ADHD) is a neurodevelopmental condition defined by symptoms of inattention, hyperactivity, and impulsivity, which impair quality of life, social, and academic outcome^1^. The condition is highly prevalent worldwide, with estimates of 5% prevalence in childhood and about 2.5% in adults^2^. Current ADHD diagnostic procedures, according to international guidelines, are built around rater-dependent assessments, including a diagnostic interview, as well as self- and third-party-reports and rating scales^3^. These are, however, subjective procedures and therefore prone to biases^4^. The limitations of current diagnostic criterion standards are widely acknowledged, and the clinical practice is criticized for mis-, over- or under-diagnosing ADHD^5^. Thus, developing biomarkers is an important field in psychiatric research to help improve the diagnostic accuracy and provide treatment guidance in the context of precision psychiatry.

Extensive research has been dedicated to exploring biomarkers in ADHD, including neuropsychological tests, electroencephalography (EEG), structural and functional brain imaging, and genetics^6,7^. However, to date, research does not support immediate clinical applications of these biomarkers for ADHD, mostly because they lack convincing accuracy and/or have limited practicality. The importance of confirming the reliability of these biomarkers through larger cohort studies that also account for sex differences has been emphasized^8^. One possible avenue to develop clinically feasible biomarkers is the development of high-dimensional digital biomarkers based on multiple clinically feasible objective measures and appropriate statistical models, including machine learning (ML)^6^.

One component of future, high-dimensional biomarkers could be based on voice assessments. The study of voice in ADHD is motivated for several reasons. First, from a neurobiological perspective, altered dopamine signaling is thought to play an important role in the pathophysiology of ADHD, amongst others related to deficits in executive functioning (EF) and motor control^9^. Moreover, dopamine is intimately involved in motor behaviors and plays a central role in vocal production^10^. Speech production is rated as one of the most complex motor behaviors, based on the coordination of more than 100 muscles, including laryngeal, supralaryngeal, and respiratory muscles. Thus, based on the hypothesis of altered dopamine signaling in ADHD, changes in speech production will possibly occur in individuals with ADHD. Secondly, EF deficits are core symptoms of ADHD and are linked to speech production. Stronger EF is associated with more accurate articulation in children and better speech performance (articulatory control and fluency of language output) in adults^11-14^.

Consistent with this, speech production deficits in ADHD have been linked to impairments in working memory and EF^15,16^. Children with ADHD, and sometimes their parents, poorly modulate voice volume, often speak louder and for longer periods and show signs of hyperfunctional voice disorder^17,18^. They have higher subglottal pressure and lower transglottal airflow, likely due to increased muscle tone in the glottis^17,19^.

Adults with ADHD show lower articulatory accuracy and slower speech rates compared to controls, with articulatory accuracy negatively correlated with symptom severity^20^. Articulation requires complex motor control dependent on self-regulation and inhibition. Lastly, recent work by Li et al. suggests that prosodic and deep-learned linguistic features can distinguish ADHD from healthy controls, achieving a classification accuracy of 0.78 ^22^.

In summary, changes in voice and speech production likely occur in ADHD and thus could serve as part of a high-dimensional biomarker. The aim of this study is to determine whether prosodic voice features recorded in brief speech tasks may allow for a differentiation of ADHD from healthy controls and subjects with other psychiatric diseases using a machine learning -based approach.

## Materials and methods

### Participants

A large heteroneous group of 563 adults were recruited at our specialized adult ADHD outpatient clinic, 387 of whom were subsequently diagnosed with ADHD (Table 1), and 100 of whom were diagnosed with other mental disorders (psychiatric controls PC) (Table S3) but did not fulfill the diagnostic criteria for ADHD. Further, 76 patients were excluded due to positive drug screenings (*n* = 55), technical deficits of the recordings (*n* = 7), or because they showed subclinical ADHD symptoms but did not fulfill the criteria for ADHD or other mental disorders after the diagnostic evaluation (*n* = 14). All participants were asked to provide voice samples before undergoing a standardized diagnostic procedure. In addition, 204 non-patient adults were recruited as healthy controls (HC) through public announcement. Healthy controls had no history of past or present neuropsychiatric conditions. All participants were aged between 18 and 59 years, and gave written informed consent (Box S1 for full inclusion/ exclusion criteria). A detailed sample description is provided in Table 1 and Table S2.

**Table 1:** Sample description.

	male	female	total
All participants	388	379	767
Age in years / mean (SD)	34.3 (10.5)	33.4 (9.8)	34.4 (10.6)
ADHD (all)	221	166	387
ATT	117	81	198
COM	104	85	189
ADHD with comorbidity	117	84	201
HC	75	129	204
PC	36	64	100
Excluded	56	20	76

*ATT*  inattentive ADHD subtype,* COM *combined inattentive and hyperactive / impulsive ADHD subtype,* HC * healthy controls,* PC * psychiatric controls

The authors assert that all procedures contributing to this work comply with the ethical standards of the relevant national and institutional committees on human experimentation and with the Helsinki Declaration of 1975, as revised in 2008. All procedures involving human subjects/patients were approved by the ethics committee of the Charité Universitätsmedizin, Berlin, Germany; the approval number is EA4/014/10. The study was registered as a clinical trial (ClinicalTrials.gov Identifier: NCT01104623).

### Diagnostic procedure

All patients were assessed by trained and licensed psychologists and psychiatrists affiliated with the specialized ADHD adult outpatient clinic of the Charité University Hospital, Berlin, Germany. A full diagnostic workup, including ADHD diagnoses, was obtained through a multi-informant, multi-method approach with psychological and medical assessments. The diagnostic procedure was structured in accordance with recommended practice of national and international guidelines^23^ and represents criterion standards for the diagnosis of ADHD in adults. It included a diagnostic interview of mental diseases according to DSM criteria, a review of client history (including developmental, medical, academic, and social background) and relevant documentation, behavioral observations, completion of self- and third party-report, and standardized ADHD-specific rating scales both for childhood and adulthood. These evaluations were completed over two sessions with up to 8 h of assessment (Table 2). To evaluate ADHD-related symptom severity, a clinical interview was performed by a senior clinician according to DSM-related criteria^23^, using the ADHD-DC scale^24^. In addition to 18 items covering inattention, hyperactivity, and impulsivity symptoms, the age of onset, symptoms related burden, general burden, and reduced social contacts were rated on 0–3-point Likert scales: 22 items in total with sum scores ranging from 0 to 66.

**Table 2:** Diagnostic procedure.

Psychiatric assessment		
	Patients	Healthy controls
Psychiatric/ psychological examination, psychiatric history	+	+
SCID I - screening	+	+
SCID I / II – diagnostic interview	+	
BDI II	+	
Medical examination	+	
Medical and psychiatric history	+	+
Blood tests, including screening for thyroid conditions	+	
Drug tests	+	+

ADHD specific assessment		
	Patients	Healthy controls
ADHD-checklist for DSM-IV ^1^	+	+
Wender Utah rating scale, short version ^2^	+	+
Semi-structured clinical interview following DSM-IV-TR	+	
Second party reports, school reports	+	

1: Diagnostic procedure according to the guidelines of the German Society for Psychiatry, Psychology and Neurology, Psychosomatics (DGPPN)^24^, 2: citation ^65^, German version ^66^;* SCID*  structured clinical interview part I/ II; BDI II – Beck Depression Inventory-II

### Voice recordings

High-quality audio recordings were obtained by a recording technician in a quiet, dedicated room free of external noise sources before each clinical evaluation, ensuring that participants and recording technicians were unaware of the diagnostic results. Prior to the actual recording, instructions for the participants were recorded and played back through an interface ( “Fast Track” Interface (MediTECH Electronic GmbH) to minimize the effects of interactions with the recording technicians and ensure a standardized recording procedure. The recording instructions included a brief rehearsal phase, during which no data was recorded. This was done to ensure that participants were familiar with the instructions and the recording process. Participants spoke into a headset microphone (C555L AKG), and signals were recorded in 16-bit/22.5 kHz sampling rate on a PC, followed by cutting and labeling procedures as preparation for the sound analysis via the “Fast Track” Interface (MediTECH Electronic GmbH).

All participants were asked to speak the following utterances: spoken single vowels and consonants, reading out single words, counting from one to ten (two trials), and free speech of around 2 min duration on a free topic, e.g. the last weekend or holidays (Table S4). Previous research indicates that voice production varies depending on the cognitive demands of the specific speech task^25,26^ .

Each clinical appointment was preceded by the standardized recording task described above. Since most participants had multiple clinical visits, multiple recordings were obtained from the same individuals using identical procedures. All recordings followed this consistent protocol, including repeated voice recordings before each follow-up appointment. For the final analysis, a maximum of three recordings per participant were included as separate data points.

From an initial 1029 voice recordings from 767 participants (including participants that were later excluded), 24 recordings were excluded due to technical problems of the recordings (e.g., low volume) or multiple recordings, i.e., more than three recordings of the same person. From the remaining 1005 recordings, 85 recordings were excluded: (a) due to positive drug screenings of the subjects (*n* = 60), because participants showed subclinical ADHD-symptoms but did not qualify for ADHD or other mental disorders (*n* = 24) or due to severe cold (*n* = 1). Thus, a total of 920 recordings were included in the classification steps, using 1 to 3 recordings per participant. A total of 71 recordings were made while patients were on medication (Methylphenidate or Atomoxetine), either prescribed prior to their initial presentation at our clinic or initiated following our diagnostic assessment.

### Feature generation and machine learning

Paralinguistic features were calculated, focusing solely on prosodic information without analyzing semantic content. Only utterances based on free speech and counting were included in the analyses. Following a *Fourier* transformation of the voice recordings^27^, paralinguistic features were calculated in two feature groups pertaining to (1) the contour of loudness and (2) the contour of speech melody (i.e. the course of F_0_)^28-31^. In brief, loudness was transformed to loudness as perceived by humans (i.e. Sone) based on the model of subjective loudness by Zwicker^32^. The transformed loudness was then averaged over 24 different time spans with durations, ranging from 0.020 s (s) to 4.032 s (Box S2). Thus, we obtained 24 contours of loudness, each representing temporal characteristics of speech sound across two orders of magnitude of time scales: from very short timespans (20 ms) informative of ‘micro prosodic’ structures up to longer time spans (4 s) capturing voice relations between words. The first derivative of all timeframes was calculated and subsequently means, standard deviations, peaks and quantiles were calculated.

For a second set of features pertaining to changes pitch, F_0_ was extracted and transformed on a logarithmic scale. The resulting values were again averaged over the same 24 different time spans (Box S2), and again after calculating the first derivative means, standard deviations, peaks and quantiles were calculated^33^. To account for statistical effects related to the dimension time of the recordings, the curves of the respective features were fitted to multiple high-dimensional vectors using proprietary code of “PeakProfiling”. In total 6145 raw features were calculated. Differences of this approach with frequently used voice analyses are given in Table 3.

**Table 3:** Comparison of general and current approach to loudness analysis.

general approach	current approach
Spectrogram employing short-time Fourier transform	Spectrogram employing short-time Fourier transform
Calculating the root-mean-square value for each time frame of the spectrogram Generating a single contour of energy over time	Calculating the loudness value (Zwicker) for each time frame of the spectrogram Generating a contour of loudness over time
	Smoothing the contour by 24 time spans ^1^, so obtaining 24 different contours
Calculating statistics of the single contour – e.g. max, min, mean, standard deviation	Calculating statistics of the 24 contours - max, min, mean, standard deviation, and higher level features derived from the slopes including the curvature, the number of peaks and valleys and the relations between them

1 time spans range between 20 ms and 4032 ms, details in Box S2 of the supplement

Prior to the classification, initial filtering was performed to eliminate features with very low (< 2.2 e -16) variance (*n* = 0) and variables with less than 100 unique values (*n* = 4), resulting in 6141 features that were included in further analyses. Random forest (RF) classifications were conducted using MATLAB Version 2023a, employing the TreeBagger algorithm. The model comprised 500 decision trees, and performance evaluation was achieved through 10-fold cross-validation repeated 100 times to ensure robustness and mitigate overfitting. Prior to classification, potential confounding variables, namely sex, age, and education, were accounted for. Each feature underwent linear regression to regress out these confounds in a cross-validation consistent manner. This involved estimating the regression model on the training fold and then applying the model to both the training and validation folds, ensuring that the residuals, which are the confound-free features, were retained for further analysis. Classification performance was quantified using the area under the receiver operating characteristic curve (AUC-ROC), referred to as AUC.

To assess the prediction capacity of the voice features regarding continuous variables such as ADHD symptom severity according to the ADHD-DC scale, but also potential confounder such as age and education, we calculated RF based regression models with 100 trees again within the cross-validation folds described above with 100 repetitions. Mean absolute error of the regression and Pearson’s correlation coefficients between actual and predicted variables were calculated as performance measures. To compare Pearson’s coefficients, we first calculated Fisher r-to-z transformations and then performed a conservative version of t-tests as proposed by Nadeau and Bengio^34^ to account for the possibility of bias related to cross-validation analyses.

To assess prediction accuracy across different subgroups (e.g., sex and age groups), we repeated all analyses in the subgroups male, female and in two age groups of similar sample size, split at the median age of 32 years. To explore differences between these subgroups, group comparisons were performed using t-tests in a similar manner as described above. To evaluate the relevance of specific features and speech tasks for the classification of ADHD, the feature importance was assessed as defined by the permutation of out-of-bag predictions as implemented in MATLAB^35^. The top ten features were extracted for further evaluation.

## Results

### Descriptive results

ADHD subjects exhibited higher symptom severity than HC and PC subjects (Table 4). Comparisons with excluded subjects remained non-significant. Within the ADHD group, symptoms in the subscale ‘inattention’ were more pronounced than in the subscale ‘hyperactivity/impulsivity’; however, the latter showed a higher variance, as indicated by a significant Levene’s test. Moreover, female ADHD participants showed more pronounced ‘hyperactivity’ than male ADHD participants. Symptom severity was similar among age groups of comparable sample sizes after a median split of age (i.e., 18–31.9 years and 32–59 years).

**Table 4:** Test psychology.

	Clinical Interview M ± SD	Inattention M ± SD	Hyperactivity/ Impulsivity M ± SD	WURS-k M ± SD	BDI - II M ± SD
ADHD (all)	40.0 ± 8.3	7.0 ± 1.7 ^2^	4.9 ± 2.5 ^2^	38.9 ± 12.9	13.3 ± 8.9
ADHD - male	40.5 ± 8.3	7.0 ± 1.7	4.7 ± 2.5*	39.4 ± 12.9	12.7 ± 8.7
ADHD - female	39.3 ± 8.3	6.9 ± 1.6	5.2 ± 2.6*	38.3 ± 12.9	14.0 ± 9.2
ADHD - ATT	35.1 ± 7.3**	7.0 ± 1.6	2.8 ± 1.6**	36.6 ± 11.6**	13.3 ± 9.0
ADHD - COM	44.7 ± 6.6**	6.9 ± 1.8	7.0 ± 1.1**	41.5 ± 13.7**	13.0 ± 8.9
ADHD < 32 y ^1^	40.2 ± 8.0	7.0 ± 1.5	4.7 ± 2.6	38.0 ± 11.8	13.7 ± 9.0
ADHD > = 32 y ^1^	39.5 ± 8.9	6.8 ± 1.8	5.0 ± 2.5	39.8 ± 13.7	12.8 ± 8.8
HC	5.2 ± 4.4***	0.5 ± 0.8***	0.5 ± 0.8***	6.6 ± 6.5***	3.2 ± 2.8***
PC	23.3 ± 12.2***	3.3 ± 2.5***	2.7 ± 2.3***	23.7 ± 13.8***	14.9 ± 8.8***
Excl. Particip.	35.9 ± 11.6	5.5 ± 2.4	4.5 ± 2.6	39.1 ± 15.4	16.5 ± 9.3

*ATT * inattentive ADHD subtype,* COM*  combined inattentive and hyperactive/impulsive ADHD subtype,* HC *  healthy controls,* PC*  psychiatric controls,* WURS-k*  Wender-Utah-rating-scale short form,* BDI*  Beck’s depression inventory; * *p* < 0.05 in comparisons of sex; ** *p* < 0.001 in comparisons ATT vs. COM; *** *p* < 0.001 in comparisons ADHD vs. HC and ADHD vs. PC; 1: Comparisons between age-groups of ADHD-participants remained non-significant; 2: Comparing the variance of inattention and hyperactivity with Levene’s test indicates higher variance in hyperactivity than in inattention

### Classification

**Fig. 1:**
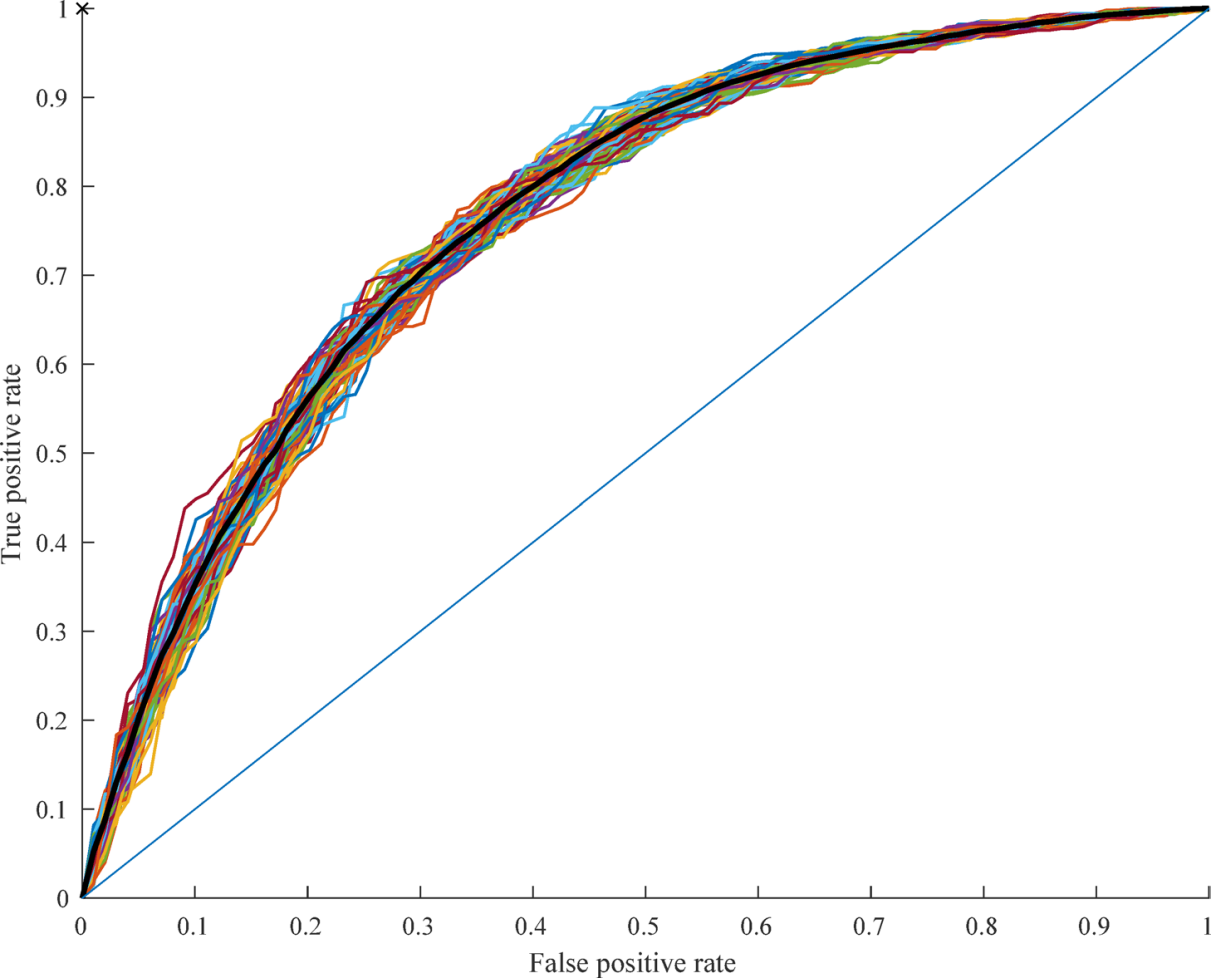
Classification ROCs for (all) ADHD patients versus healthy controls (801 recordings). Random-forest classification, 10-fold cross-validation; AUC = 0.77.

RF classification was calculated, controlling for effects of age, sex, and education based on voice features. Cross-validated out-of-sample accuracies are provided in Table 5; Fig. 1. The classification over the complete sample (ADHD patients versus HC based on 801 recordings) resulted in an AUC of 0.77, which is invariant to classification threshold. Repeating the classification with speaker-stratified cross-validation produced largely consistent results (AUC differences ranging from 0.00 to 0.03 in subanalyses, detailed in Supplementary Table S6), with no change in AUC for the complete sample.

**Table 5:** Classification ADHD vs. HC.

Group		AUC	Precision	Recall	F1
All		0.77	0.60	0.75	0.66
Male		0.70	0.51	0.90	0.66
Female		0.83	0.71	0.78	0.74
one recording/ participant		0.76	0.63	0.73	0.67
excl. stimulants		0.78	0.64	0.77	0.70
excl. comorbid ADHD		0.78	0.70	0.73	0.72
ADHD, subtype ATT		0.79	0.73	0.75	0.74
ADHD, subtype COM		0.80	0.72	0.75	0.73
Age 18–31	All	0.80	0.68	0.79	0.73
(n = 385 recordings)	Male (*n* = 202)	0.75	0.53	0.72	0.62
	Female (*n* = 183)	0.87	0.82	0.81	0.81
Age 32–59	all	0.72	0.55	0.75	0.63
(n = 416 recordings)	Male (*n* = 193)	0.59 ^1^	0.50	NaN	0.50
	Female (*n* = 223)	0.74	0.57	0.73	0.64

*ATT *  inattentive ADHD subtype,* COM*  combined inattentive and hyperactive/impulsive ADHD subtype,* HC *  healthy controls, 1: This calculation should be interpreted with caution, as it suffers from class imbalance with only 30 HC

### ADHD-subtype, sex, age

The classification showed a largely similar performance in subjects with combined symptoms (i.e., including hyperactivity and impulsivity) compared to predominantly inattentive subjects. Analyzing both sexes separately pointed toward a higher AUC in females than in male participants (*t* = 3.3, *p* = 0.001). A higher AUC in female subjects was also evident when analyzing separate age groups (age < 32: *t*_*male/ female*_ = 2.2, *p* = 0.03; age > 32: *t*_*male/ female*_ = 2.3, *p* = 0.03, Table 5) and in the non-comorbid ADHD sample (male: AUC = 0.68; female AUC = 0.79; *t* = 3.3, *p* = 0.001). Comparisons of age groups indicated superior classification performance in the younger sample (AUC = 0.80 vs. AUC = 0.71; *t* = 2.0, *p* = 0.04), with the highest performance in young female participants (AUC = 0.86).

### Comorbidity, psychiatric controls, number of speech samples, medication

The classification of a subsample of ADHD subjects with no comorbidity resulted in a comparable AUC of 0.78. Calculating the classification of ADHD participants against a psychiatric control (PC) group, which presented for the evaluation of ADHD, but did not meet the clinical criteria of ADHD, showed a lower prediction accuracy (AUC = 0.60, Precision = 0.91, Recall = 0.61, F1 = 0.71). Notably, the PC group still exhibited elevated symptoms of ADHD as indicated by elevated symptom scores in the clinical interview of ADHD compared to HC (mean sum score psychiatric controls (PC): 23 vs. 5 in HC; mean sum score 40 in ADHD, Table 4). Subgroup details (age, sex) are provided in Supplementary Table S5.

Furthermore, utilizing a single recording per subject yielded a classification performance that was nearly equivalent to that achieved with multiple recordings (0.76 vs. 0.77). Excluding subjects who took ADHD medication (methylphenidate, atomoxetine) before the voice recording did not alter the classification performance, however the number of affected recordings was modest (*n* = 71; 8.9% of the analyzed sample).

### Correlations of symptom severity using random forest regression

**Fig. 2:**
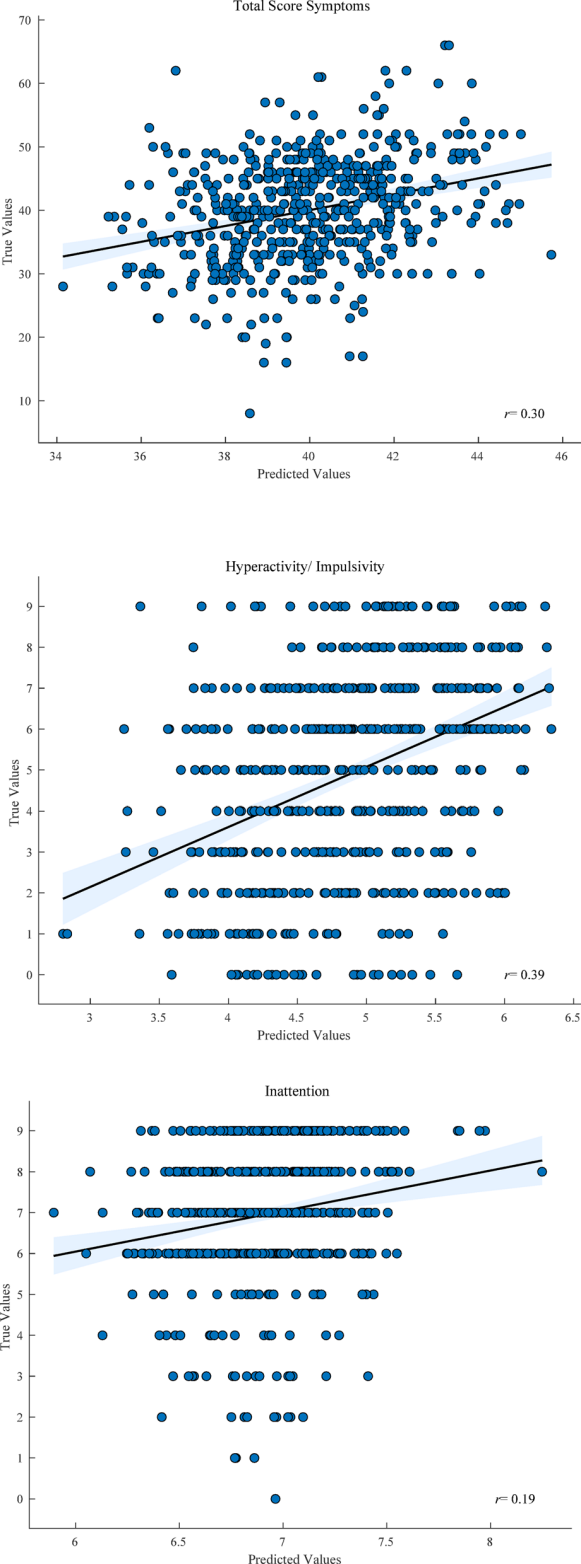
Correlation of true and predicted symptom severity scores (from voice features) in ADHD subjects across scales of the ADHD Clinical Interview: total score symptoms, hyperactivity, inattention. Mean slope and 95% confidence intervals depicted; *r* = Pearson’s correlation coefficient; all *p* < 0.001.

Correlations of true and predicted symptoms based on voice features were calculated using RF regressions. The results are given in Table 6; Fig. 2. Analyses performed in the ADHD group only indicated a higher association of true and predicted hyperactive/ impulsive symptoms than inattention symptoms (*r*_hyper_ = 0.39; *r*_inattention_ = 0.19; comparison: *t* = 3.6, *p* < 0.001). Extending the analysis of ADHD symptom severity and voice features to all participants (ADHD, PC, HC) yielded a higher association of true and predicted symptoms (*r* = 0.45, *t* = 14.7, *p* < 0.001), in particular in female subjects (*r*_female_ = 0.49; r_male_ = 0.29; comparison *t* = 3.4, *p* < 0.001).

**Table 6:** Correlation coefficients of true and predicted clinical variables.

	Group	*n*	*r*	t	MAE
ADHD clinical interview^1^	All	876	0.45	14.7	12.5
ADHD clinical interview^1^	Female	457	0.49	12.1	12.9
ADHD clinical interview^1^	Male	419	0.29	6.3	12.0
Hyperactivity	All	876	0.36	11.6	2.3
Inattention	All	876	0.39	12.9	2.6
Education	All	920	0.33	10.5	0.7
Age (years)	All	920	0.52	18.5	7.2
Beck’s depression inventory^1^	All	701	0.24	6.6	7.1
ADHD clinical interview^1^	adhd	554	0.30	6.7	6.3
Hyperactivity	adhd	554	0.39	9.2	2.0
Inattention	adhd	554	0.19	3.9	1.3

Pearson’s correlation coefficients are given based on RF regression;* MAE*  mean absolute error, *BDI*  Beck’s depression inventory; *n* = number of voice samples; 1: total symptoms score

*p*-value for all analyses < 0.001

### Confounding variables

To analyze confounders likely influencing voice analysis, we performed predictions of sex, age, and education (highest educational level) using RF classification and RF regression as appropriate in the entire sample (ADHD, PC, HC). Sex was classified with AUC = 0.98. The correlations of true and predicted age (*r* = 0.52, *t* = 18.5, *p* < 0.001) and education (*r* = 0.33, *t* = 10.5, *p* < 0.001) likewise pointed to relevant associations with voice features and were thus included in all analyses as confounders of no interest. Moreover, sex (*t* = 5.4, *p* < 0.001), age (*t* = -1.7, *p* = 0.09) and education (*t* = 5.9, *p* < 0.001) were associated with ADHD status and therefore included as covariates of no interest, as described above. Given concerns about changes in results due to confound leakage^36^, we re-ran all analysis without confound removal. The results were largely identical, with changes of AUC between 0.00 and 0.05.

Since depression has been linked to changes of loudness in past research^37^, we calculated associations of depression scores with voice features (*r* = 0.24, *t* = 6.6, *p* < 0.001). However, depression scores were not related to ADHD symptom severity; thus, we did not include it as covariate in the main analysis. Including depression severity as covariate in a secondary analysis did not change the results.

### Analysis of feature importance for the prediction of ADHD

The ten most important features were equally (50%) distributed to free speech (2 min), based on loudness changes over time frames between 0.02 and 0.252 s, and changes of F_0_ in the counting task in timeframes of 0.02 to 0.1 s.

## Discussion

In the context of a growing interest in analyzing voice as a potential biomarker of mental health^38^, to the best of our knowledge, we have conducted the first study to investigate prosodic features in a large, heterogeneous sample of adults with ADHD. The results support and extend previous findings of paralinguistic abnormalities in ADHD and point toward the possibility of predicting ADHD in unseen individuals, particularly in early adulthood. The classification showed good prediction accuracy in differentiating ADHD from HC, and this was confirmed in associations of true and predicted symptom severity.

Previous studies in the emerging field of automatic assessments of mental disorders using speech were restricted to mood and psychotic disorders^38^, and they showed comparable prediction accuracies to those reported in this study. For instance, depression can be predicted from (paralinguistic) voice features with an accuracy of about 80% ^39^. The prediction of schizophrenia has been successful using linguistic features, such as semantic relatedness, differentiating between individuals with schizophrenia or those at a high risk to develop acute symptoms and healthy controls^40^. Studies that have investigated the prediction of ADHD from biological signals other than voice point towards a similar performance of approaches based on neuropsychological assessment^41^, EEG-measures^42^, questionnaires^43^ or resting state fMRI^44^. Thus, the findings in this study are comparable to previous research using voice to predict mental disorders and research relying on other biological signals to predict ADHD.

Moreover, correlations between true and predicted symptom scores, both in the whole sample and within the ADHD group, indicate that voice features were able to predict ADHD symptom severity. The continuous association of voice-features and ADHD severity, in turn, may help to interpret the lower prediction accuracy of ADHD from a PC group. The PC group presented for a diagnostic workup to rule out ADHD and showed markedly elevated ADHD symptoms as compared to HC. After an extensive diagnostic procedure, the participants in this group were not diagnosed with ADHD but with various other mental disorders, mostly affective disorders. Thus, since the classifier seems to be sensitive to lower levels of ADHD symptoms as indicated by the RF-regression analyses of symptom severity above, the classification is likely less suited to differentiate between ADHD-suspect subjects with lower symptoms and ADHD subjects who fullfil the clinical criteria. Moreover, the PC group was heterogeneous with regards to mental disorders, and this may contribute to the limited differentiation from ADHD. Future studies with larger sample size may be able to predict ADHD with higher precision and better differentiate ADHD participants from individuals with low or moderate ADHD symptoms. Likewise, other studies investigating potential biomarkers of ADHD point to a restricted differentiation from PC, such as studies of neuropsychological tests in combination with actigraphy^45^ or studies using MRI^46^.

A strength of the current approach is related to a robust classification performance of ADHD in the presence of comorbid mental disorders. The classifier showed a similar performance to differentiate ADHD from HC with and without comorbidity. Regarding the role of comorbidity, we additionally controlled the analyses for depression symptom severity with no changes in the AUC. Of note, participants with severe clinical comorbidity, such as schizophrenia or severe affective disorders were excluded from the analysis. Adult ADHD patients frequently present with latent or lifetime comorbid conditions^47^, thus compromising the diagnostic accuracy of ADHD in clinical practice^48^. Overall, the limited influence of comorbidity in this study is encouraging in terms of potential clinical applications; replication of our results pending. Since our data point to a relatively robust accuracy particularly in younger and female participants - possibly independent of comorbidity - automated voice analysis could evolve as a valuable addition to support the diagnostic process, considering marked diagnostic challenges amongst others due to comorbidity in this patient group^49^.

Evaluating the prediction accuracy of inattentive and hyperactivity/impulsivity symptom scores, the RF-regression analyses pointed to a higher correlation of true and predicted symptoms of hyperactivity than of inattention in the ADHD group. Importantly, we noted a higher variance of the hyperactivity/impulsivity symptoms than of inattention in the ADHD group, as indicated by a significant Levene’s test. Thus, the higher correlation of true and predicted hyperactivity/impulsivity scores may be explained by a better ability for the RF regression to learn from a sample with a larger variance. Using the whole sample (i.e., ADHD, PC, HC), inattention and hyperactivity subscales showed similar prediction scores of true and predicted symptoms (with similar variance of both variables in this group). In line with this, the higher correlation of true and predicted hyperactivity/impulsivity symptoms did not translate into a significantly better prediction of the subgroup with combined (inattention and hyperactive/impulsive) symptoms.

With regard to emotion regulation or personality traits as a second important symptom cluster of ADHD, prior research has pointed to associations of personality traits and prosody of speech^37,50^, including associations of impulsivity and F_0_/ jitter^51^, that are related to the features in this study. Nilsen et al.^18^ reported higher speech volumes and pitch to be related to decreased inhibitory control, a key component of impulsivity. Moreover, a higher motor activity in ADHD, may result into higher subglottal pressures that have been reported in children with ADHD^17^. In summary, a relation of prosodic speech abnormalities and ADHD traits such as deficits in EF or hyperactivity/impulsivity seems plausible and should be evaluated in more detail in future research.

Discussing secondary findings, sex was classified with a high accuracy (AUC = 0.98) using voice features. The high accuracy is in line with recent studies on predicting sex from prosodic voice features^52^. Furthermore, our data indicate a better classification performance of ADHD in female over male subjects, and this pertained to subsamples with different age-groups and non-comorbid ADHD-subjects. To some extent the sex related differences may be explained by slightly higher hyperactivity scores in female ADHD, but other measures of symptom severity or comorbidity did not differ between sexes. Thus, we assume that the voice features vary between male and female subjects and are possibly more pronounced in female ADHD subjects, though this must be investigated in future studies including the underlying biological mechanisms. One possible explanation may be related to sex specific dopamine receptor expression and functioning^53^; given the important role of dopamine for voice production^10^, sex specific dopamine function may pertain to voice. Previous studies on voice in psychiatric disorders have mostly not differentiated between sex due to limited sample size, but this has been requested^38^.

With respect to age, the voice features in this study were able to predict age with a comparable if slightly lower performance to other approaches predicting age from prosodic features^54^. Research on changes of prosody in aging indicates higher scores of hoarseness, instability and breathiness in higher age^55^, reflected by a diminished harmony to noise ratio^56^. Moreover, with increasing age, the subglottal pressure decreases in line with a decrease in overall muscle mass, and subjects compensate for this with increased expiratory airflow^57^. In study with children, increased hoarseness has been reported in ADHD^17,58^ as well as an increased subglottal pressure and decreased airflow^17^. Thus, if healthy subjects in older age may show increased hoarseness, which could also occur in ADHD, a possible difference in prosodic features due to hoarseness may decrease with older age and thus explain the decreased classification performance in older subjects. Since the finding on hoarseness in ADHD have been reported in children, our hypotheses related to age should be tested in adult populations in future research.

Stimulants have been reported to impact voice and prosody in a few small studies, including reports of lower F_0_, increased jitter^59^, and increased hoarseness^60^. In our study, we did not note differences with regard to classification accuracy and intake of stimulants, however the subsample with intake of stimulants was relatively small.

To understand which features are relevant to differentiate ADHD from HC, the analyses of feature importance firstly point to changes in loudness as an important differentiating feature. This finding supports previous research that identified voice anomalies in ADHD, frequently pertaining to loudness^15^. Breznitz et al. reported differences in temporal speech patterns and physical features of vocalization in boys with ADHD^61^ compared to PC (reading disabilities) and HC. Children with ADHD showed increased loudness, and children with combined ADHD type were louder, and showed lower F_0_^16,62^. We assume that subtle changes in voice based on higher subglottal pressure in ADHD^17^ may result in relevant changes of loudness to differentiate ADHD from healthy subjects.

Secondly, feature importance data indicate that speech test selection plays an important role in the classification of ADHD. Five of the ten features with the highest feature importance were related to counting (from one to ten) and five to free speech. It has been shown in previous research, that speech task (complexity) is related to vocal features, e.g. vocal variability differs between spontaneous speech and reading aloud^63^ and a larger vocal variability was observed in a picture description task than in recalling autobiographical memories^64^. Since EF mirrored by task complexity is related to voice and EF is known to be compromised in ADHD, a combination of speech tasks with varying complexity may be relevant for the differentiation of ADHD from HC and should be included in future trials.

In summary, we were able to differentiate ADHD patients from HC using voice features with good results in early adulthood and in female subjects. Strengths of the present study include the large, heterogeneous sample, the assessment of HC and PC and the application of criterion diagnostic procedures. The study’s limitations include an imbalance in subgroup sizes, which may have particularly affected performance in male participants. Additionally, the exact time of day for each recording was not documented, which may have influenced the results.

We detected ADHD associated vocal patterns that likely reflect a disorder related vocal hyperfunction possibly related to altered dopamine signaling. These vocal patterns may be related to personality traits such as impulsivity, impaired EF, and changes in motor functioning pertaining to speech in ADHD. Neurobiologically, these traits are related to dopamine, which is thought to play an important role in the pathogenesis of ADHD^9^. Replication pending, a strength of voice-based features to predict ADHD might be the lack of impact from psychiatric comorbidity, that frequently occurs in ADHD and complicates the diagnostic process.

Given the feasibility and low cost to record and analyze voice, we see a potential value for future clinical application as a digital biomarker and encourage further investigation. A larger study sample including a larger PC group will be necessary to achieve a better differentiation of ADHD from PC with low or moderate ADHD symptoms. However, the clinical value of a voice-based screening might be in supporting the clinician to include potential differential diagnoses rather than excluding differential diagnoses based on one test. In future research, the classification performance may increase with the addition of distinct speech features, such as speech pauses and the utilization of linguistic measures such as verbal fluency. Taken together, we consider voice analysis a promising avenue to support the diagnostic process in adult ADHD.

## Electronic supplementary material

Below is the link to the electronic supplementary material.

Supplementary Material 1

## Data Availability

The data that support the findings of this study are available from PeakProfiling GmbH with certain restrictions. The data were used under license for this study. Please contact co-author JL with requests. The code of the analyses was written in MATLAB and is available from the corresponding author (GP) upon request.
