# Supplementary material for: Exploring voice as a digital phenotype in adults with ADHD
Source: Sci Rep. 2025 May 24;15:18076. doi: 10.1038/s41598-025-01989-x (PMC12103603; doi:10.1038/s41598-025-01989-x)
Supplement: Supplementary file 1 — Supplementary Material 1 [file 41598_2025_1989_MOESM1_ESM.pdf]

Supplementary Information

Data storage: Data are stored at Charité Universitaetsmedizin Berlin, Department of Psychiatry, Campus Benjamin Franklin, Berlin, Germany.

Box S1 Inclusion and Exclusion criteria

Inclusion Criteria:

1. Age 18-59.
2. Written informed consent.
3. Adult ADHD / Non-ADHD/ other disorders according to the fourth version of the Diagnostic and Statistical Manual for Mental Disorders (DSM-IV)
4. Normal vocal functioning, negative history for voice or laryngeal disorders.
5. Currently euthyroid condition; in case of past history of thyroid disease and need for current pharmacological treatment, patient needs to be on stable medication for at least 4 weeks.

Exclusion Criteria:

1. Severe mental conditions such as schizophrenia and psychotic disorders not otherwise specified, severe depressive episode, severe substance use disorder.
2. Positive drug tests.
3. Severe medical condition such as epilepsy.

Box S2 Length of feature timewindows in ms:

20, 25, 32, 40, 50, 63, 79, 100, 126, 159, 200, 252, 318, 400, 504, 635, 800, 1008, 1270, 1600, 2016, 2540, 3200, 4032

Table S1 Distribution of recordings

	**male**	**female**	**total**
All recordings	505	501	1005
ATT	167	123	290
COM	149	134	283
ADHD with comorbidity	175	129	304
ADHD with stimulants	32	39	71
Healthy Controls	79	149	228
Psychiatric Controls	43	76	119
Excluded	66	19	85

ATT = inattentive ADHD subgroup; COM = combined inattentive and hyperactive / impulsive ADHD subgroup; HC = healthy controls

Table S2 Education

	No school certificate	High School Certificate	High school advanced certificate (German Abitur)	Further education/ university
All participants	10	268	257	225
ATT	1	76	66	55
COM	6	81	65	37
HC	0	38	72	94
PC	1	41	31	27
Excluded	2	32	23	12

ATT = inattentive ADHD subtype; COM = combined inattentive and hyperactive / impulsive ADHD subtype; HC = healthy controls; PC = psychiatric controls

Table S3 Diagnostic distribution of clinical controls

**Diagnosis**	**n**
Addiction disorder (e.g. games)	13
Schizophrenia	3
Bipolar disorder	1
Depressive episode	49
Dysthymia	12
OCD, PTSD (ICD-10 Chapter 4)	35
Specific phobia	16
Social phobia	11
Anorexia nervosa, Bulimia nervosa	3
Borderline PD	9
Narcissistic PD	13
Avoidant-restrictive PD	10
other PD	17

OCD = Obsessive compulsive disorder; PTSD = post-traumatic stress disorder; PD = Personality disorder

Table S4 Recorded utterances from each participant

**Utterance**	**mean duration (s)**
Free speech	120
Counting 1-10, two trials	22
Reading out single words	20
Sounds (3 sec) a: i: u: ç f s n	21
Recorded material per participant	183

Table S5 Classification ADHD vs Psychiatric controls

Group	AUC	Precision	Recall	F1
All	0.60	0.91	0.61	0.71
Male	0.57	0.94	0.62	0.71
Female	0.53	0.87	0.56	0.63
Age 18 – 31	0.60	0.96	0.53	0.64
Age 32 – 59	0.61	0.92	0.61	0.70

Table S6 Classification ADHD vs HC with speaker stratified cross-validation

Group		AUC	Precision	Recall	F1
all		0.77	0.82	0.86	0.83
male		0.70	0.82	0.94	0.87
female		0.82	0.84	0.74	0.78
**Age 18 – 31**	all	0.79	0.83	0.80	0.81
**(n = 385 recordings)**	male (n = 202)	0.73	0.80	0.87	0.83
	Female (n = 183)	0.85	0.90	0.70	0.78
**Age 32 – 59**	all	0.70	0.81	0.88	0.84
**(n = 416 recordings)**	male (n = 193) [1]	0.56	0.84	0.96	0.89
	female (n = 223)	0.71	0.77	0.78	0.76

HC= healthy control; 1: This calculation should be interpreted with caution, as it suffers from class imbalance with only 30 HC
